# Chronic Vestibular Hypofunction Is Associated with Impaired Sleep: Results from the DizzyReg Patient Registry

**DOI:** 10.3390/jcm12185903

**Published:** 2023-09-11

**Authors:** Benedict Katzenberger, Fiona Brosch, Stéphane Besnard, Eva Grill

**Affiliations:** 1 Institute for Medical Information Processing, Biometry and Epidemiology (IBE), Faculty of Medicine, LMU Munich, 81377 Munich, Germany; 2 Pettenkofer School of Public Health, 81377 Munich, Germany; 3 Laboratory of Cognitive Neurosciences, UMR7291, Team Pathophysiology and Therapy of Vestibular Disorders, Centre National de la Recherche Scientifique (CNRS), Aix Marseille University, 13331 Marseille, France; 4 Research Group on Vestibular Pathophysiology, Centre National de la Recherche Scientifique (CNRS), Unit GDR2074, 13331 Marseille, France; 5 German Center for Vertigo and Balance Disorders, LMU University Hospital, LMU Munich, 81377 Munich, Germany

**Keywords:** balance, posture, sleep disorder, compensation, vertigo, dizziness, pittsburgh sleep quality index, vestibular hypofunction

## Abstract

Temporary or permanent vestibular hypofunction has been hypothesized to affect circadian rhythm, sleep, and thermoregulation. Chronic or long-term vestibular disorders such as unilateral vestibular hypofunction may have an even greater negative impact on sleep quality than acute vestibular problems. This study examines self-reported sleep quality, as assessed by the Pittsburgh Sleep Quality Index (PSQI), and its association with vestibular symptom duration in a group of patients with vestibular disorders. We used data from the cross-sectional DizzyReg patient registry of the German Center for Vertigo and Balance Disorders outpatient clinic. Vestibular diagnoses were ascertained based on the International Classification of Vestibular Disorders. A total of 137 patients were included (60% female, mean age 55.4 years, standard deviation, SD, 16.7). The mean PSQI total score was 6.3 (SD = 3.2), with 51% reporting overall poor sleep quality. Patients who had vertigo for two years or longer reported significantly poorer global sleep quality (63% vs. 37%, *p* = 0.021) and significantly more difficulties with sleep latency (79% vs. 56%, *p* = 0.013) and sleep efficiency (56% vs. 34%, *p* = 0.022). The association of poor sleep quality with a longer duration of vertigo remained significant after multivariable adjustment. Further research should investigate the interaction of vestibular disorders, sleep, and their potential mechanisms.

## 1. Introduction

The vestibular system is the brain’s most direct way of sensing movements of the body. Three vestibular sensory organs, the semicircular canals, detect angular accelerations of the head in three spatial planes, while two additional organs, the maculae, detect linear accelerations of head and body movements. The brain simultaneously integrates and combines this vestibular information about gravity-related motion and body position with information from neck and body proprioception. Apart from the most obvious functions for gait and stance control, the dense integration of vestibular perception into functional brain pathways indicates several equally important but less acknowledged roles. The brain integrates vestibular sensory information at different levels. Vestibular nuclei in the brainstem behave as a hub for these multisensory inputs. They are also at the origin of functional pathways relevant not only for postural but also for gaze fixation, orthostatic neurovegetative regulation, hormonal functions on the hypothalamo-hypophysis axis, motion-related emotion, and high-level cognitive functions, including awareness of the own body position and orientation [1]. Temporary or permanent vestibular hypofunction impairs cognitive functioning, such as required for spatial memory and pathfinding [2], and it has been hypothesized to affect circadian rhythm, sleep and thermoregulation [3,4,5].

In this context, sleep and the vestibular system have distinct relevance. Gentle rocking, i.e., vestibular stimulation, is used in almost all cultures to help babies fall asleep. Rocking was also found to promote sleep in mice with normal vestibular function [6]. Disturbed sleep impacts almost all physiological and mental functions [7] and is a risk factor for, e.g., metabolic, cognitive, and endocrine pathologies. The close relationship between sleep and the vestibular system is highlighted by specific findings in pathologies which disrupt sleep. Sleep deprivation contributes to dizziness and disturbed visuospatial performance [8], as does fragmented sleep due to sleep apnea obstructive syndrome [9]. Sleep apnea is also associated with poor postural control [10] and decreased cervical vestibular evoked myogenic potential responses, possibly reflecting hypoxia-related abnormalities in brainstem pathways [11]. Experimental sleep deprivation and insomnia showed distinct effects on vestibular parameters, such as altered ocular vestibular-evoked myogenic potential reflexes [12] and visuospatial perception and attention [13]. Moderate sleep deprivation reduced spatial memory performance and hippocampal cell activity [14].

Several studies in animals and humans point to a vestibular regulation of circadian rhythms via the suprachiasmatic nucleus or a vestibular regulation of sleep through orexinergic neurons and the locus coeruleus. Circadian rhythms were changed in rats with experimentally induced permanent bilateral vestibular loss [15]. In humans, long-term bilateral vestibular loss was associated with a misalignment of body temperature and rest-activity cycles and reduced sleep efficiency [5]. There is also increasing evidence that vestibular impairment may affect the different domains of sleep, specifically sleep quality and duration. A large population-based study found that sleep duration per night was either significantly shortened or very prolonged in persons with vestibular vertigo [16]. Also, looking at specific vestibular pathologies, patients with benign paroxysmal positional vertigo (BPPV) [17] or vestibular migraine [18] reported significantly worse sleep quality than healthy controls. This detrimental effect on sleep was even more pronounced in patients with a longer history of BPPV [17]. Moreover, these combined findings lead to the hypothesis that chronic or long-term vestibular disorders such as unilateral vestibular hypofunction have an even greater negative impact on sleep quality than acute vestibular problems [19]. Conversely, interventions of vestibular rehabilitation improved sleep parameters in patients with unilateral vestibular hypofunction [20].

This study aimed to examine the quality of self-reported sleep in a group of patients with vestibular diseases characterized according to high clinical standards. In particular, we were interested in whether the duration of vestibular disorders was associated with sleep quality, sleep duration and other clinically relevant aspects of sleep.

## 2. Materials and Methods

### 2.1. Data Collection Procedures and Participants

We used cross-sectional patient records from the DizzyReg patient registry of the outpatient clinic of the German Center for Vertigo and Balance Disorders (DSGZ) at the University Hospital Munich, Ludwig-Maximilians University, Germany. This hospital is a tertiary medical center with 2000 beds and 82,000 inpatients per year. The DSGZ sees around 3000 outpatients a year. Since 2015, DizzyReg has collected data on consenting patients from DSGZ [21]. DizzyReg is an ongoing project that collects data prospectively at one point, i.e., the diagnoses are recorded simultaneously with all other variables. Also, retrospective information enters the registry, such as medical history, duration of symptoms, and previous healthcare utilization for vertigo. Patients are eligible for the registry if they present at the DSGZ for a diagnostic workup and if informed consent is given by the patient or their legal surrogate. Patients with terminal illnesses, cognitive impairment, or language skills not sufficient for understanding the questionnaires are not part of the registry. At the clinic, patients receive comprehensive neuro-otological examinations and tests during one entire day, e.g., video head impulse tests or calorimetry, as needed for a definite diagnosis and are also asked to complete questionnaires on symptoms, previous health services utilization, and patient-reported outcomes such as functioning, sleep quality and health-related quality of life. The registry draws information from the clinical workplace system, questionnaires, or medical records, including discharge letters. Vestibular diagnoses that enter the registry are routinely ascertained by experienced neuro-otologists based on the International Classification of Vestibular Diseases and the diagnostic standards of the Bárány Society [22]. For this study, we included all consecutive, newly admitted patients aged 18 years or older from April 2022 until August 2022. We did not formally calculate the required sample size a priori. This was due to the exploratory nature of the analyses. For the current study, we defined the type of vertigo as peripheral if the underlying diagnosis was associated with defects in the peripheral vestibular system (including BPPV and Meniere’s disease), as central if the diagnosis was referred to defects in the central nervous system and as functional if vertigo was primarily driven by psychological factors.

The ethics committee of the medical faculty at Ludwig-Maximilians-University (LMU) Munich, Germany, approved the study (approval code 414-15). All participants gave written informed consent. The registry works in accordance with the Declaration of Helsinki principles.

### 2.2. Measures

Sleep quality and sleep disturbances over the last four weeks were assessed retrospectively during the patient’s stay at the clinic using the Pittsburgh Sleep Quality Index (PSQI) [23]. This patient-reported questionnaire has 19 questions that can be combined to a total score ranging from 0 to 21 and 7 sleep difficulty sub-scores (subjective sleep quality, sleep latency, using sleep medication, sleep duration, habitual sleep efficiency, sleep disturbances, and daytime dysfunction due to sleepiness) with higher score values indicating worse sleep. Each sub-score ranges from zero to three points, with zero points indicating no sleep problems and three points indicating severe problems. Adding up the scores of each component yields the global PSQI score, which indicates global sleep quality. In line with the literature [23], we categorized participants with a global score of 5 or below as good sleepers and participants with global scores above five as poor sleepers. Sub-scores were defined as the absence of the respective sleep problem if the sub-score equaled zero or as the presence of problems if the sub-score was greater than zero.

We defined the duration of vestibular disorders based on participants’ self-reports and verified this by medical records. Categorization as ‘two years or less’ or as ‘more than two years’ was motivated by statistical power considerations using the median as the threshold.

Covariates were selected according to the literature for the most relevant predictors of either exposure and/or outcome, which are not instrumental variables [24] and therefore included age in years, gender, alcohol consumption, smoking, and multimorbidity.

The presence or absence of alcohol use was assessed for the last seven days. We calculated the average amount of alcohol consumed in grams per day based on self-reported type and amount of alcoholic beverages consumed on the previous weekend and the last working day before the visit at the DSGZ. Smoking status was categorized as either “never” if a participant indicated “no” or as “current” if a participant indicated “yes” to the question “Do you currently smoke?” The category “former” indicated that a participant was not smoking but affirmed the question, “Did you ever smoke?” The presence of two or more chronic diseases was defined as multimorbidity [25] based on a list of 12 health conditions (Supplemental Table S1).

### 2.3. Statistical Analyses

We calculated unadjusted summary statistics for the overall sample and separately for patients with longer and shorter duration of vestibular disorders. We report mean values, standard deviations for continuous variables, and absolute and relative frequencies for categorical values. To examine bivariate associations, we used one-way ANOVA for continuous variables and Fisher’s Exact Test for categorical variables.

To analyze the association between the duration of vestibular disorders and sleep quality, we used multiple linear regression models. Regression diagnostics included the adjusted R^2^ for goodness of fit of the model, the studentized Breusch-Pagan test for heteroscedasticity and Q-Q plots for homogeneity of the residuals. We present the fully adjusted model with all included covariates and the respective adjusted R-squared, indicating the goodness of fit of the model.

We used R version 4.2.2 and RStudio for all analyses with a two-sided *p*-value of <0.05, defined as statistically significant.

## 3. Results

A total of 137 patients were included: 60% female, mean age 55.4 years (standard deviation, SD = 16.7), 30% with peripheral vestibular disease, 23% with central vestibular disease, 28% with functional vertigo, and 20% with other diagnoses including, e.g., polyneuropathy and orthostatic vertigo. The mean PSQI total score was 6.3 (SD = 3.2), with 51% reporting overall poor sleep quality. Patients with vestibular disorders for two years or longer reported significantly higher total scores, i.e., worse sleep. Sociodemographic characteristics are shown in Table 1.

Overall, 90% of patients with vertigo or dizziness reported poor sleep quality, 67% reported problems with sleep latency, 11% reported frequently needing sleep medication, 38% reported problems with sleep duration, 46% reported difficulties with sleep efficiency, 95% reported sleep disturbance, and 89% reported daytime sleepiness due to poor sleep. Patients who had vertigo for two years or longer reported significantly poorer global sleep quality (63% vs. 37%, *p* = 0.021) and significantly more difficulties with sleep latency (79% vs. 56%, *p* = 0.013) and sleep efficiency (56% vs. 34%, *p* = 0.022). Participants slept on average 6.9 h per night (median 7.0 h), with 11% of all patients sleeping less than 6 h. Table 2 shows details on sleep domains stratified by duration of vestibular disorders. Global sleep quality was marginally higher in patients with central vestibular disease than peripheral vestibular disease (mean PSQI total score 7.34 vs. 5.91, *p* = 0.214). Differences between types of vertigo did not reach statistical significance for either the global score or any of the subscales.

The results of the adjusted multivariable model (Table 3) are based on complete cases (*n* = 101). Patients with missing values of the global PSQI score were significantly older but did not differ otherwise from those with complete scores. The global PSQI scores were significantly higher by an average of 1.4 points in patients with vestibular disorders for two years or more, indicating poorer overall sleep quality. Men had better sleep quality than women. The presence of multimorbidity was an independent risk factor for poor sleep.

## 4. Discussion

This study of patients with vestibular disorders and other related conditions demonstrated that long-term vestibular pathology of two-year duration or longer was associated with poorer sleep quality. This association was independent of other risk factors for poor sleep that might have played a role, namely multimorbidity, age and gender. The duration of symptoms also had a negative effect on sleep latency and sleep efficiency.

Epidemiological studies show a high prevalence of insomnia worldwide, with up to 10% of adults with severe symptoms and an additional 20% reporting poor sleep quality [26]. As hypothesized, the global sleep quality of patients in our study was well below the expected average. A total of 51% of our participants with vertigo reported very poor or fairly poor sleep, which is also well above the average of 23 to 25% of the adult population [27,28] reported from representative samples in Germany. We could show that sleep problems increased with increasing duration and chronicity of the vestibular symptoms, with 63% of patients reaching or exceeding the threshold of 6 points on the PSQI. This percentage is in line with previous results from patients with chronic dizziness [29].

To start with a well-investigated component of sleep quality, sleep duration, there is sufficient evidence that sleep duration is associated with morbidity, with chronically shorter sleep predicting poor health outcomes and shorter lifespans [30]. We found that over 30% of patients with shorter symptom duration and over 45% of patients with longer symptom duration slept less than 7 h, with 11% of all patients sleeping even less than 6 h, confirming the findings from a large representative US American study [16]. Similarly, insomnia is often associated with difficulty initiating sleep, which reduces sleep efficiency because the increased time spent in bed does not contribute to satisfying, restorative sleep. In our study, patients with longer symptom duration needed, on average, 5 min more to fall asleep, with 79% of them reporting that they needed 30 min or more and that this decreased their daytime functioning. In contrast, a large European study reported that only an average of 16% of adults from the UK, Germany and Italy had similar problems with sleep latency [31], clearly adding evidence to the hypothesis that vestibular problems are associated with latency. This increase in sleep latency has also been reported, for example, in migraineurs with a mean latency difference of almost 10 min compared with people without migraines [32]. As migraine may have a vestibular component, and as patients with vestibular migraine were part of our sample, the association seems similarly plausible.

Our results indicate quite dramatically that chronic vertigo symptoms have a distinct role in sleep mechanisms, although causal pathways are still incompletely understood. The vestibular system is essential to autonomous regulation, including blood pressure, heart rate variability [33], and hormonal homeostasis, e.g., regarding thyroid hormones [34]. Sleep also interacts with sympathetic regulation and endocrine function, e.g., thyrotropin [35]. Chronic dizziness and vertigo may be a sign that the brain has not been able to compensate for the vestibular impairment adequately. Vestibular compensation typically occurs after an acute, often unilateral vestibular pathology and can be seen as a remodelling process of the hippocampus, visual cortex, and cerebellum [36]. Vestibular compensation is, therefore, an essential component of successful recovery. As a result, corticosteroid treatment for acute vertigo may worsen sleep disturbance due to its side effects. Therefore, the balance of benefits and side effects of corticosteroid therapy is debatable when considering the role of sleep-triggered neuroplasticity in vestibular compensation processes. Chronic lack of sleep decreases neuroplasticity. It has been shown for other sensory organs that sleep modulates the structure of the visual cortex [37], auditory learning involves a consolidation phase during sleep [38], and sleep is needed to reorganize and manifest olfactory experiences made during the day [39]. Vestibular impairments seem to alter important sleep parameters through their postulated non-photonic circadian action, i.e., independently of the day-night cycle [15]. Also, vestibular inputs influence the activity of orexinergic neurons and the locus coeruleus in the hypothalamus, which are related to sleep regulation [3]. Thus, vestibular impairment may disrupt neuroplasticity and reinforce a vicious cycle of disturbed sleep and lack of vestibular compensation. Arguably, this will be more pronounced the longer a vestibular impairment remains undiagnosed and underlines the need for timely diagnosis, treatment and rehabilitation of vertigo and dizziness. In addition, there is now a broad consensus that insomnia should always be treated independently in any pathology, even if it is a symptom or other comorbidity [40]. Thus, sleep should be monitored in vestibular disease, and vestibular function should be assessed concurrently with sleep problems. This is particularly important for older adults with dizziness or vertigo, as both sleep problems and dizziness are often considered inevitable in older adults. Conversely, independently of vestibular problems, age does not appear to be associated with sleep problems [41], as confirmed by the adjusted analysis of our study.

Strengths of this study include the standardized phenotyping of an established clinical registry and the valid state-of-the-art diagnostic workup of patients with vestibular disorders presenting at a large tertiary academic centre.

The small sample size may be considered a limitation of our study. Nevertheless, we could show statistically significant, relevant, and plausible differences between groups to test our main hypothesis. In addition, self-reporting sleep problems has often been mentioned as a limitation compared to polysomnography, reported as the gold standard of quantitative sleep analysis. However, self-reporting using the PSQI is a valid and established method because the questionnaire is less invasive and less likely to produce volunteer bias. Finally, this study is cross-sectional and is therefore not able to establish causal associations. Still, we could verify symptom duration retrospectively by medical records, thus increasing the validity of the exposure variable and adding a temporal component to the analyses.

## 5. Conclusions

Our results add further evidence to the hypothesis that the negative long-term consequences of vestibular problems on health might be partly explained by their interaction with sleep, probably also mediated by sympathetic regulation and endocrine pathway disruption. Future research should look more closely into the interaction of vestibular disorders, sleep and the potential mechanisms, including hormonal action.

## Figures and Tables

**Table 1:** Sociodemographic characteristics of 137 patients with vertigo and dizziness seen at an academic tertiary care center, stratified by duration of vestibular disorders. Higher values of global sleep quality (PSQI = Pittsburgh Sleep Quality Index) indicate poorer sleep quality.

		Total	<2 Years	≥2 Years	*p*-Value ^1^
N (%)		137	69 (50.4)	64 (46.7)	
Age (mean, SD)		55.42 (16.65)	53.20 (17.88)	57.59 (15.49)	0.134
Female		82 (59.9)	41 (59.4)	37 (57.8)	0.990
Global sleep quality, PSQI (mean, SD), *n* = 110		6.27 (3.18)	5.44 (2.83)	7.18 (3.37)	0.004
Smoking	current	20 (15.0)	9 (13.8)	10 (15.6)	0.888
	former	53 (39.8)	27 (41.5)	24 (37.5)	
	never	60 (45.1)	29 (44.6)	30 (46.9)	
Alcohol within the last seven days		65 (48.1)	34 (50.0)	30 (46.9)	0.853
Alcohol consumption/g per day [median, IQR] *n* = 65		11.43 [3.00, 25.71]	11.78 [4.09, 24.29]	10.00 [2.86, 22.86]	0.749
Multimorbidity present		39 (30.2)	18 (28.1)	19 (31.1)	0.862
Type of vertigo	Central	31 (22.6)	16 (23.2)	15 (23.4)	0.676
	Functional	38 (27.7)	16 (23.2)	20 (31.2)	
	Other	27 (19.7)	13 (18.8)	12 (18.8)	
	Peripheral	41 (29.9)	24 (34.8)	17 (26.6)	

^1^ ANOVA, Fisher’s Exact Test. SD = standard deviation, IQR = interquartile range.

**Table 2:** Detailed sleep characteristics of 137 patients with vertigo and dizziness seen at an academic tertiary care center, stratified by duration of vestibular disorders. Higher values of sleep quality indicate worse sleep quality.

	Total	<2 Years	≥2 Years	*p*-Value ^2^
N (%)	137	69 (50.4)	64 (46.7)	
Global sleep quality ^1^, mean (SD), *n* = 110	6.27 (3.18)	5.44 (2.83)	7.18 (3.37)	0.004
Global sleep quality ^1^: poor sleepers, *n* (%)	56 (50.9)	22 (38.6)	32 (62.7)	0.021
Sleep problems				
Sleep quality, *n* (%)	122 (90.4)	61 (89.7)	57 (90.5)	1
Sleep latency, *n* (%)	87 (67.4)	36 (56.2)	48 (78.7)	0.013
Sleep latency, mean (SD), *n* = 132	23.61 (39.89)	18.22 (18.76)	23.57 (28.26)	0.207
Sleep medication, *n* (%)	14 (10.6)	3 (4.5)	10 (16.4)	0.053
Sleep duration < 7 h, *n* (%)	51 (38.3)	20 (30.8)	29 (45.3)	0.128
Sleep duration, mean (SD), *n* = 133	6.89 (1.37)	7.11 (1.53)	6.70 (1.17)	0.090
Habitual sleep efficiency, *n* (%)	61 (46.2)	22 (33.8)	35 (55.6)	0.022
Sleep disturbance, *n* (%)	115 (95.0)	61 (93.8)	52 (96.3)	0.851
Daytime dysfunction, *n* (%)	115 (88.5)	58 (89.2)	55 (88.7)	1

^1^ Global scores were calculated for 110 patients with complete data for all scores. ^2^ ANOVA, Fisher’s Exact Test. SD = standard deviation, IQR = interquartile range.

**Table 3:** Analysis of the association of duration of vestibular disorders with global sleep quality (*n* = 101) adjusted for covariates. Higher values of global sleep quality (PSQI = Pittsburgh Sleep Quality Index) indicate poorer sleep quality.

		Estimated PSQI Score (95% Confidence Interval)
(Intercept)		**5.14 (3.07; 7.21)**
Duration of vestibular disorders	**≥2 years**	**1.34 (0.15; 2.53)**
Age		0.02 (−0.02; 0.06)
Gender	**Male**	**−1.27 (−2.51; −0.03)**
Smoking	Current	Reference
	Former	−0.51 (−1.84; 0.82)
	Never	0.25 (−1.45; 1.94)
Alcohol consumption within the last seven days	Yes	−1.14 (−2.35; 0.07)
Multimorbidity	**Yes**	**1.94 (0.59; 3.29)**
Adjusted R-squared		0.19

## Data Availability

This project uses data from the DizzyReg registry. The variable list is detailed in Supplementary Materials Table S2. The dataset is available from the DizzyReg by application.

## Supplementary Materials

The following supporting information can be downloaded at https://www.mdpi.com/article/10.3390/jcm12185903/s1, Table S1: Assessment of comorbidities in the DizzyReg patient registry; Table S2: Detailed variable list.
