# Supplementary material for: Chronic Vestibular Hypofunction Is Associated with Impaired Sleep: Results from the DizzyReg Patient Registry
Source: J Clin Med. 2023 Sep 11;12(18):5903. doi: 10.3390/jcm12185903 (PMC10531914; doi:10.3390/jcm12185903)
Supplement: Supplementary file 1 [file jcm-12-05903-s001.zip › Supplemental Table S1 Assessment of comorbidities in the DizzyReg patient registry.pdf]

Chronic vestibular hypofunction is associated with impaired sleep: Results from the DizzyReg patient registry

Benedict Katzenberger [1,2,*], Fiona Brosch [1,2], Stéphane Besnard [3,4] and Eva Grill [1,5]

[1] Institute for Medical Information Processing, Biometry and Epidemiology (IBE), Faculty of Medicine, LMU Munich, 81377 Munich, Germany
[2] Pettenkofer School of Public Health, 81377 Munich, Germany
[3] Laboratory of Cognitive Neurosciences, UMR7291, Team Pathophysiology and Therapy of Vestibular Disorders, Aix Marseille University, Centre National de la Recherche Scientifique (CNRS), 13331 Marseille, France
[4] Research Group on Vestibular Pathophysiology, Centre National de la Recherche Scientifique (CNRS), Unit GDR2074, 13331 Marseille, France
[5] German Center for Vertigo and Balance Disorders, LMU University Hospital, LMU Munich, 81377 Munich, Germany
* Correspondence: benedict.katzenberger@med.uni-muenchen.de

Supplemental Table S1 Assessment of comorbidities in the DizzyReg patient registry

- Asthma
- Chronic obstructive pulmonary disease or emphysema
- Chronic bronchitis
- Neurological disease (multiple sclerosis, epilepsy)
- Liver disease (e.g. cirrhosis)
- Cardiac insufficiency
- Angina pectoris, coronary sclerosis
- Cardiac arrhythmia
- Hay fever
- Kidney disease
- Inflammatory joint disease
- Hypertension
