# Supplementary material for: Chronic Vestibular Hypofunction Is Associated with Impaired Sleep: Results from the DizzyReg Patient Registry
Source: J Clin Med. 2023 Sep 11;12(18):5903. doi: 10.3390/jcm12185903 (PMC10531914; doi:10.3390/jcm12185903)
Supplement: Supplementary file 1 [file jcm-12-05903-s001.zip › Supplemental Table S2 Detailed variable list.pdf]

Chronic vestibular hypofunction is associated with impaired sleep: Results from the DizzyReg patient registry

Benedict Katzenberger [1,2,*], Fiona Brosch [1,2], Stéphane Besnard [3,4] and Eva Grill [1,5]

1 Institute for Medical Information Processing, Biometry and Epidemiology (IBE), Faculty of Medicine, LMU Munich, 81377 Munich, Germany
2 Pettenkofer School of Public Health, 81377 Munich, Germany
3 Laboratory of Cognitive Neurosciences, UMR7291, Team Pathophysiology and Therapy of Vestibular Disorders, Aix Marseille University, Centre National de la Recherche Scientifique (CNRS), 13331 Marseille, France
4 Research Group on Vestibular Pathophysiology, Centre National de la Recherche Scientifique (CNRS), Unit GDR2074, 13331 Marseille, France
5 German Center for Vertigo and Balance Disorders, LMU University Hospital, LMU Munich, 81377 Munich, Germany
* Correspondence: benedict.katzenberger@med.uni-muenchen.de

Supplemental Table S2 Detailed variable list

Variable	Label	Type	Categories and Transformation
**Sociodemographic characteristics**			
age	Age in years	Numeric	
gender	Patients' gender	Categorical	1, Male<br>2, Female
period_cat	Duration of vertigo and dizziness symptoms	Categorical	0, Two years or less<br>1, More than 2 years
smoking_status	Smoking status	Categorical, Transformed	1 ("Current"), nicotine = 1<br>2 ("Former"), nicotine_yesno = 1 & nicotine = 0<br>3 ("Never"), nicotine_yesno = 0
nicotine	Do you currently smoke?	Categorical	0, No<br>1, Yes
nicotine_yesno	Did you ever smoke?	Categorical	0, No<br>1, Yes
alcohol	Did you consume at least one alcoholic beverage within the last 7 days?	Categorical	0, No<br>1, Yes
alcohol_amount	Average amount of alcohol consumed in grams per day	Numeric	
morbidity	Multimorbidity (>= 2 comorbidities present)	Categorical	0, No<br>1, Yes
**Sleep difficulty sub-scores**			
PSQI_TOTAL	Global sleep quality, index	Numeric	
PSQI_TOTAL_bin	Global sleep quality	Categorical, Transformed	0 ("Good sleepers"), PSQI_TOTAL <= 5<br>1 ("Poor sleepers"), PSQI_TOTAL > 5
PSQI_SLPQUAL_bin	Problems with sleep quality	Categorical, Transformed	0 ("No problems"), psqi_07 = 0<br>1 ("Present problems"), psqi_07 > 0
PSQI_LATEN_bin	Problems with sleep latency	Categorical, Transformed	0 ("No problems"), psqi_02 <=15.0 & psqi_06a =0<br>1 ("Present problems"), psqi_02 >15.0 | psqi_06a >0
PSQI_MEDS_bin	Medication needed to fall asleep	Categorical, Transformed	0 ("No meds taken"), psqi_08 = 0<br>1 ("Meds taken"), psqi_08 > 0

Continued on page 2

Supplemental Table S2 Detailed variable list (continued)

Variable	Label	Type	Categories and Transformation
**Sleep difficulty sub-scores**			
PSQI_DURAT_bin	Sleep duration	Categorical, Transformed	0 (“At least 7 hours”), psqi_04 >= 7<br>1 (“Less than 7 hours”), psqi_04 < 7
TMP_HSE	Ratio of time actually slept and total time spend in bed	Numeric, Transformed	(psqi_04 / diff(psqi_01, psqi_03)) * 100
PSQI_HSE_bin	Sleep efficiency,	Categorical, Transformed	0 (“No problems”), TMP_HSE >= 85<br>1 (Present problems”), TMP_HSE * 100 < 85
PSQI_DISTB_bin	Sleep disturbance	Categorical, Transformed	0 (“No disturbance”), all psqi_06a to psqi_06j = 0<br>1 (“Any disturbance”), any psqi_06a to psqi_06j > 0
PSQI_DAYDYS_bin	Day disfunction due to sleepiness	Categorical, Transformed	0 (“No problems”), psqi_09 = 0 & psqi_10 = 0<br>1 (“Present problems”), psqi_09 > 0 | psqi_10 > 0
**Sleep difficulty - questions**			
psqi_01	What time did you usually go to bed in the evening during the last four weeks?	Numeric	
psqi_02	In the last four weeks, how long did it usually take you to fall asleep (in minutes)?	Numeric	
psqi_03	What time did you usually get up during the last four weeks?	Numeric	
psqi_04	How many hours per night did you actually sleep in the last four weeks?	Numeric	
psqi_05	How many hours per day spent napping?	Numeric	
psqi_06a	How many times in the last four weeks have you slept poorly because you could not fall asleep within 30 min?	Categorical	0, Not during the past four weeks<br>1, Less than once a week<br>2, Once or twice a week<br>3, Three or more times a week
psqi_06b	How many times in the last four weeks have you slept poorly because you woke up in the middle of the night or early in the morning?	Categorical	0, Not during the past four weeks<br>1, Less than once a week<br>2, Once or twice a week<br>3, Three or more times a week
psqi_06c	How many times in the last four weeks have you slept poorly because you had to get up to go to the bathroom?	Categorical	0, Not during the past four weeks<br>1, Less than once a week<br>2, Once or twice a week<br>3, Three or more times a week

Continued on page 3

Supplemental Table S2 Detailed variable list (continued)

Variable	Label	Type	Categories and Transformation
**Sleep difficulty - questions**			
psqi_06d	How many times in the last four weeks have you slept poorly because you had trouble breathing?	Categorical	0, Not during the past four weeks<br>1, Less than once a week<br>2, Once or twice a week<br>3, Three or more times a week
psqi_06e	How many times in the last four weeks have you slept poorly because you had to cough or snored loudly?	Categorical	0, Not during the past four weeks<br>1, Less than once a week<br>2, Once or twice a week<br>3, Three or more times a week
psqi_06f	How many times in the last four weeks have you slept poorly because you were too cold?	Categorical	0, Not during the past four weeks<br>1, Less than once a week<br>2, Once or twice a week<br>3, Three or more times a week
psqi_06g	How many times in the last four weeks have you slept poorly because you were too warm?	Categorical	0, Not during the past four weeks<br>1, Less than once a week<br>2, Once or twice a week<br>3, Three or more times a week
psqi_06h	How many times in the last four weeks have you slept poorly because you had bad dreams?	Categorical	0, Not during the past four weeks<br>1, Less than once a week<br>2, Once or twice a week<br>3, Three or more times a week
psqi_06ii	How many times in the last four weeks have you slept poorly because you had pain?	Categorical	0, Not during the past four weeks<br>1, Less than once a week<br>2, Once or twice a week<br>3, Three or more times a week
psqi_06j	How many times in the last four weeks have you slept poorly because for other reasons?	Categorical	0, Not during the past four weeks<br>1, Less than once a week<br>2, Once or twice a week<br>3, Three or more times a week
psqi_07	Overall, how would you rate your sleep quality over the past four weeks?	Categorical	0, Very good<br>1, Fairly good<br>2, Fairly bad<br>3, Very bad
psqi_08	During the last four weeks, how often have you taken medicine (prescribed or "over the counter") to help you sleep?	Categorical	0, Not during the past four weeks<br>1, Less than once a week<br>2, Once or twice a week<br>3, Three or more times a week
psqi_09	During the last four weeks, how often did you have difficulty staying awake, for example, while driving, eating, or during social events?	Categorical	0, Not during the past four weeks<br>1, Less than once a week<br>2, Once or twice a week<br>3, Three or more times a week
psqi_10	During the last four weeks, have you had any problems doing the usual daily tasks with enough drive?	Categorical	0, No problems<br>1, Few problems<br>2, Some problems<br>3, Major problems
